# Stem cell therapy, smoking and obesity

**DOI:** 10.1590/S1516-31802005000400001

**Published:** 2005-07-07

Most people tend to emphasize unusual events that occur in their own lives or professional careers. In giving such emphasis to these events when speaking to an inattentive listener, an individual may pass on an image that is very different from the reality of his behavior in the past. In medical activity, difficult clinical diagnoses, unusual test results and undesirable reactions to medications leave a much greater impression than do easy diagnoses, test results that are compatible and coherent with the clinical state, and successful treatments. Despite mocking journalists for whom news only consists of "man bites dog" stories and not the opposite, we doctors have the same habit of emphasizing infrequent occurrences and downplaying day-to-day activities of the profession and research of general use.

Even today, in some medical schools, scientific research is confounded with the description of rare cases. In other words, critical analysis of day-to-day activities is transformed into the presentation of clinical cases that would be more appropriate for a museum collection. They have some use, but their priority is much less important. The same thing occurs today in defining the priorities for public health, with a preference for everything that causes repercussion in the communication media. In an analogous manner, in news about medicine and health, only therapeutic methods such as "transplants" or "stem cells" merit being news. Incidentally, two of our colleagues in services in two different states have even released to the lay press the result (note the singular) from one case using stem-cell therapy on a patient with Chagas' disease cardiopathy and another from a patient with a cerebrovascular event.

This interpretation of medicine and health as spectacles gives rise to allocations of public and private funds to services with little usefulness. It is rare for the municipal, state and federal administrators to analyze the statistics from their own diligent health information services. To give an example, it can be shown that, even though prostate cancer has been indicated as the most important problem for the male population, instigated by large numbers of publicity campaigns, there were 15 deaths due to cardiovascular disease for each death due to prostate cancer in the state of São Paulo in 2002. Likewise, there were 10 deaths due to cardiovascular disease among women for each death resulting from breast cancer, which has been the target of large numbers of official and non-governmental campaigns. These data can easily be obtained from the Ministry of Health's internet site for health statistics: http://www.datasus.gov.br.

Cardiovascular diseases are the most important problem for medical attention today, and smoking and obesity are among their principal risk factors. A study was conducted by the Epidemiological Surveillance Center of the Health Department of the State of São Paulo* in 2001 and 2002, to assess the prevalence of cardiovascular risk factors in the municipality of São Paulo, utilizing the methodology of cross-sectional population studies.^1^ This study may only be one among many others, but it shows the time sequence of these important risk factors by making a comparison with the results from a similar study carried out in 1987 (Table 1).

**Table 1:** Percentage prevalence ratios (and 95% confidence intervals) of cardiovascular risk factors in the municipality of São Paulo, over a fifteen-year period. Adapted from Coutinho et al.^1^

		1987	2001-2002
Smoking	Men	41.8 (37.5-46.2)	25.5 (22.7-28.2)
	Women	30.6 (27.7-33.4)	19.8 (17.5-22.2)
Obesity	Men	6.1 (4.0-8.2)	12.4 (10.5-14.4)
	Women	9.3 (7.5-11.1)	15.0 (13.0-17.1)

Smoking, measured as the proportion of current smokers, underwent a notable reduction in prevalence both among men (-39%) and among women (-35%).

This can be proven by comparing the age group distribution of smokers between 1987 (Figure 1)^2^ and 2001-2002 (Figure 2). There was a reduction at all ages. In the 15-29 age group, in which the smoking addiction begins, the reduction was the sharpest: -49% among men and -53% among women.

**Figure 1:**
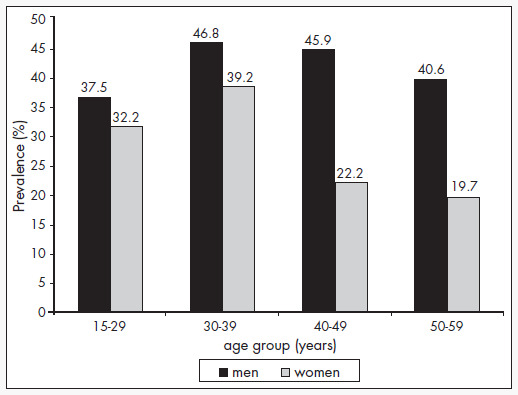
Prevalence of smoking (current smokers) by sex and age group in the municipality of São Paulo, 1987. Adapted from Rego et al.^2^

**Figure 2:**
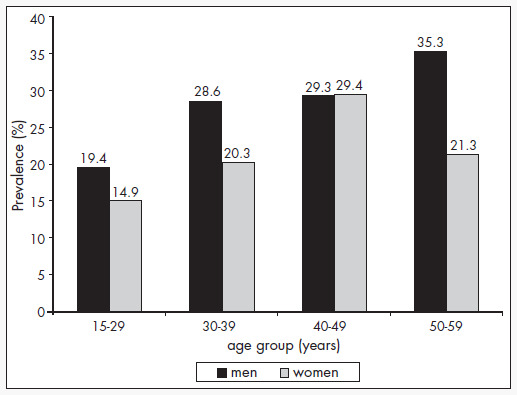
Prevalence of smoking (current smokers) by sex and age group in the municipality of São Paulo, 2001-02. Adapted from Coutinho et al.^1^

Although data on former smokers is not available, to assess the rate of quitting, it was also observed in 2001-2002 that the lower prevalence was always in younger age groups, except among women in the 40-49 age group. In other words, lower prevalence occurred in the generation that was born in the 1950s, in comparison with individuals born in earlier decades.

Another important finding was the reaffirmation of the increasing obesity in the city, up by 100% for men and 60% for women, which could cause an increase in diabetes over the short to medium term. Unlike the smoking data, the increase in obesity has already been confirmed empirically in other population-based studies, and it has already been reported in the pages of *Diagnóstico & Tratamento* [where this article was first published in Portuguese].

Perhaps the lesson that we must accept is that, over a 15-year period, there have been significant and radical changes in two factors of the greatest importance, smoking and obesity, which may alter the profile of cardiovascular diseases in our population. This would indeed be a topic to be discussed in the daily routine of health departments, medical schools and specialist societies, and should have repercussions in the press. And this is regardless of whether it is "good news" like the reduction in smoking or the "bad news" of increasing obesity.

As for stem-cell therapy, let us hope that serious and competent research, carried out with an adequate number of participants will indicate that it is useful, performed in the same way as the research on myocardiopathy currently being funded by the Ministry of Health.

And, for smoking, we need to increase the present regulatory control and provide treatment for nicotine dependents. The debate on obesity is an open matter all around the world, and the solutions need to come more from the dynamics of society than from genetics or hormones.

## Footnotes

*
*This study was only published online, at the internet address of the Alexandre Vranjac Epidemiological Surveillance Center (Centro de Vigilância Epidemiológica "Alexandre Vranjac"- http://www.cve.saude.sp.gov.br/), under the title: "Pesquisa: Fatores De Risco Para Doenças Crônicas" (Research: risk factors for chronic diseases). The authors were Ana Paula Coutinho, Adriana Bouças Ribeiro, Africa Isabel de la Cruz Prez Neuman, Ana Maria Aratangi Pluciennik, Artur Jaques Goldfeder (editor), Luiz Francisco Marcopito (coordinator), Marco Antonio de Moraes, Mirian Matsura Shirassu, Raimundo Sicca, Rodolfo Brumini, Sérgio Sans Fins Rodrigues, Sidney Federman, Ana Maria Sanches, Maria Aparecida Pacheco, Maria Célia Medina; all from the Division of Chronic Non-transmittable Diseases of the Epidemiological Surveillance Center, Health Department of the State of São Paulo (CVE-SES/SP).*
